# 4-Hydroxynonenal Contributes to Fibroblast Senescence in Skin Photoaging Evoked by UV-A Radiation

**DOI:** 10.3390/antiox10030365

**Published:** 2021-02-28

**Authors:** Audrey Swiader, Caroline Camaré, Paul Guerby, Robert Salvayre, Anne Negre-Salvayre

**Affiliations:** 1 Inserm U1048, Institute for Metabolic and Cardiovascular Diseases, 31432 Toulouse, France; audrey.swiader@inserm.fr (A.S.); caroline.camare@inserm.fr (C.C.); paul.guerby@gmail.com (P.G.); rsalvayre@gmail.com (R.S.); 2 Department of Biochemistry, University of Toulouse III—Paul Sabatier, 31062 Toulouse, France; 3 Department of Gynecology/Obstetrics, Toulouse University Hospital, 31300 Toulouse, France

**Keywords:** 4-hydroxynonenal, fibroblasts, senescence, vimentin, carnosine, skin, UV-A, photoaging

## Abstract

Solar ultraviolet A (UV-A) radiation promotes a huge variety of damages on connective tissues and dermal fibroblasts, including cellular senescence, a major contributor of skin photoaging. The mechanisms of skin photoaging evoked by UV-A partly involve the generation of reactive oxygen species and lipid peroxidation. We previously reported that 4-hydroxynonenal (HNE), a lipid peroxidation-derived aldehyde, forms adducts on elastin in the skins of UV-A irradiated hairless mice, possibly contributing to actinic elastosis. In the present study, we investigated whether and how HNE promotes fibroblast senescence in skin photoaging. Dermal fibroblasts of skins from UV-A-exposed hairless mice exhibited an increased number of γH2AX foci characteristic of cell senescence, together with an accumulation of HNE adducts partly colocalizing with the cytoskeletal protein vimentin. Murine fibroblasts exposed to UV-A radiation (two cycles of 15 J/cm^2^), or HNE (30 µM, 4 h), exhibited senescence patterns characterized by an increased γH2AX foci expression, an accumulation of acetylated proteins, and a decreased expression of the sirtuin SIRT1. HNE adducts were detected on vimentin in cultured fibroblasts irradiated by UV-A or incubated with HNE. The HNE scavenger carnosine prevented both vimentin modification and fibroblast senescence evoked by HNE in vitro and in the skins of UV-A-exposed mice. Altogether, these data emphasize the role of HNE and lipid peroxidation-derived aldehydes in fibroblast senescence, and confirm the protective effect of carnosine in skin photoaging.

## 1. Introduction

Solar ultraviolet (UV) radiation is a main cause of premature skin aging (photoaging), characterized by a loss of skin tone, mottled skin pigmentation, deep wrinkle appearance, and sagging, with possible pathological complications including actinic elastosis, actinic keratosis and cancers [1,2,3,4]. While mutagenic UV-B rays are almost completely absorbed by epidermis, UV-A radiation, which is the most abundant component of solar UV radiations at the earth surface, penetrates deeply into the skin, affecting both the epidermis and the dermis [2,3,4,5]. Both UV-A and UV-B can indirectly damage DNA by generating reactive oxygen species (ROS), which progressively alter local antioxidant defenses and promote oxidative stress, a key-player of the photoaging process [6,7,8,9,10]. UV-induced cellular damages are a main trigger of cellular senescence in photoaged skin, particularly for dermal fibroblasts, which rapidly acquire a senescence-associated secretory phenotype (SASP) upon exposure to UV radiation [10].

Skin dermal fibroblasts are poorly proliferative, a source of extracellular matrix components (ECM), and highly involved in the control of structural and mechanical skin properties [10,11]. Skin fibroblasts continuously adapt to photoaging damages, which progressively promote their dysfunction and ECM remodeling [10,11,12,13]. The number of senescent fibroblasts is increased by photoaging [13], while many phenotypic changes related to intrinsic and extrinsic aging have been identified in these cells, such as persistent DNA damages, chromosome instability, or telomere shortening [10,11]. Several senescence biomarkers could be observed, including the expression of senescence-associated β-galactosidase (SA-βGal) [14], as well as an increased percentage of cells exhibiting γH2AX foci in their nuclei [15,16]. Alterations of the cytoskeleton occur [17], possibly resulting from changes in intermediate filaments such as vimentin, a cytoskeletal protein linked to aging [18,19,20]. Vimentin expression is increased in senescent cells [19], and old fibroblasts exhibit vimentin modifications by glycation and AGEs, or by lipid peroxidation products resulting from the oxidation of polyunsaturated fatty acids by UV-radiation [10,17,18,20]. Lipid peroxidation-derived aldehydes, such as 4-hydroxynonenal (HNE), acrolein, or malondialdehyde (MDA) [21,22,23], accumulate in photoaged skin [24,25,26,27,28,29]. These agents could play a role in cellular senescence, particularly in foam cells and endothelial cells in atherosclerosis [30], in trophoblasts during accelerated placental aging [31], and on skin fibroblast aging at least in vitro [24].

In a previous study, we reported that HNE and acrolein generated in the skin of hairless mice repeatedly exposed to UV-A radiation contribute to skin photoaging by forming adducts on extracellular matrix components in the dermis, particularly on elastin, with possible implication in solar elastosis [32]. Based on this previous study, the aim of the present article was to investigate whether and how HNE contributes to fibroblast senescence elicited by UV radiation. Interestingly, carnosine, an efficient carbonyl and HNE-scavenger [33,34], was able to prevent the modification of elastin [32], and we show here that it is also able to reduce skin fibroblasts senescence elicited by UV-A radiation in vivo, and prevent HNE-induced senescence of cultured skin fibroblasts.

## 2. Materials and Methods

### 2.1. Antibodies and Reagents

Cell culture reagents were from Invitrogen Life Technologies (Thermofisher). Anti-HNE-Michael adduct antibodies were from Oxis Research (#24327) for immunofluorescence studies, and from Invitrogen (#MA5-27570), for immunoprecipitation experiments. The anti-vimentin monoclonal antibody was from Abcam (#ab92547). Anti-γH2AX (#9718S), anti-SIRT1 (#9475S), anti-acetylated-Lysine (#9441S), and secondary anti-mouse and anti-rabbit HRP-conjugated antibodies were from Cell Signaling Technology. Anti-ubiquitin antibody was from Santa Cruz Biotechnology (#sc-8017). 3-(4,5 dimethylthiazol-2-yl)-2,5-diphenyltetrazolium bromide (MTT), L-Carnosine, 2-phenylindole dihydrochloride (DAPI), and anti β-actin antibody were from Sigma-Aldrich. Secondary Alexa Fluor antibodies 488 and 546 were from Life Technologies.

### 2.2. Cell Culture and UV-A Treatment

Murine skin fibroblasts (strain 129/SV) were grown in DMEM Glutamax culture medium supplemented with 10% fetal bovine serum (FBS) and antibiotics (100 U/mL penicillin, 100 mg/mL streptomycin) in a 5% CO_2_ humidified incubator, at 37 °C. Twenty-four hours before the experiments, cells were starved in serum-free medium, as indicated.

Before UV-A exposure, the medium was removed and replaced by 2 mL HBSS. Fibroblasts were exposed twice to UV-A rays (each exposure up to 15 J/cm^2^, with a 24 h delay between the two exposures) (Bio-Spectra UV lamp, 365 nm, Vilbert-Lourmat, Torcy, France). When indicated, fibroblasts were preincubated for 18 h with carnosine (100 µM), after which the medium was discarded and replaced by HBSS before exposing cells to UV-A. At the end, the HBSS medium was removed and replaced by DMEM medium containing 1% FBS and, when indicated, carnosine. Alternatively, fibroblasts were incubated with HNE (30 µM in HBSS, 4 h), with or without carnosine (100 µM); then, cells were rinsed in HBSS, incubated in DMEM medium containing 1% FBS. At the indicated times, cells were rinsed twice with PBS, and stored at –80 °C until use, or fixed in paraformaldehyde (PFA) 4% in PBS, for immunofluorescence and confocal imaging. The cell viability of fibroblasts exposed to UV-A or treated by HNE was evaluated by the MTT test assay [32].

### 2.3. Immunofluorescence and Confocal Imaging

#### 2.3.1. Murine Skins

Immunofluorescence and confocal imaging studies were done on murine skin paraffin samples from our previous study [32]. Briefly, this study was carried out on albino hairless mice Skh:hr-1 (8 weeks old, Charles River Laboratories), and four conditions (five animals/condition), had been set up, i.e., one control group (non-irradiated mice), one group of mice daily exposed to UV-A radiation (20 J/cm^2^ daily, up to 600 J/cm^2^), one group treated with polyethylene glycol (PG) (solvent for carnosine) and exposed daily to UV-A, and one group treated by carnosine (1% in PG) and exposed to UV-A (PG and carnosine were spread over the back at the end of UV-A exposure). After animal sacrifice, the skins from mouse backs were recovered and a sample was embedded in paraffin for immunohistological analysis [32] and present article. More precise experimental conditions are detailed in [32]. The experimental protocol (N°12/1048/10/13) was conducted in accordance with French legislation and the local ethical committee for animal experiments [32].

Serial 3 µm skin sections were incubated with primary anti-HNE-Michael adduct antibody (#24327, Oxis Research), anti-vimentin antibody or anti-γH2AX antibody before Alexa Fluor secondary antibody staining. Nuclei were stained with DAPI (1 µg/mL). Slides were analyzed using a Zeiss LSM 780 confocal microscope. Controls were done on unexposed skin sections or unstimulated fibroblasts.

#### 2.3.2. Murine fibroblasts

After exposure to UV-A or HNE, fibroblasts were washed with PBS and fixed in PFA 4% in PBS for 10 min. After blocking with PBS containing 5% bovine serum albumin for 45 min, cells were incubated with the primary anti-HNE-Michael adduct antibody Oxis Research (#24327) or the anti-vimentin antibody (#92547, Abcam), followed by Alexa Fluor-488 or Alexa Fluor-546 conjugated antibodies.

### 2.4. Western Blot Analysis and Immunoprecipitation

Fibroblast protein extracts were used for western blot studies as indicated, using β-actin as control for equal protein loading [32]. After solubilization in lysis buffer (10 mM TRIS pH 7.5, 1% Triton X-100, 1% Sodium deoxycholate, 0.1% SDS, 150 mM NaCl, 5 mM NaF + protease/phosphatase inhibitors cocktail), the protein content was determined by the Bradford technique, as indicated by the manufacturer. The protein extract (40 µg) was separated by SDS-PAGE and transferred to a PVDF membrane (Immobilon, Millipore). After blocking in 5% nonfat milk, membranes were blotted overnight at 4 °C with primary antibodies (1:1000). Membranes were revealed by chemiluminescence after incubation with appropriate horseradish peroxidase-conjugated secondary antibodies (1:5000), using ECL substrate (Chemidoc Touch, Biorad). For immunoprecipitation experiments, 1 mg of total protein extract was incubated with 2 µg of anti-vimentin antibody overnight at 4 °C, followed by 2 h precipitation at 4 °C with protein A-sepharose coated beads (GE Healthcare). The beads were washed three times with lysis buffer, resuspended with loading buffer, boiled for 5 min, and subjected to western blot analysis to be revealed with anti-HNE antibody.

### 2.5. Statistical Analysis

The results are expressed as mean ± SEM from at least three independent experiments. For the normally distributed data, Student’s *t*-test was used; otherwise, nonparametric Mann-Whitney *U*-test was employed. Statistical calculations were carried out using the software Graphpad Prism, version 6.01 (Graph Pad Software Inc., San Diego, CA, USA). Values of *p* < 0.05 were considered significant.

## 3. Results

### 3.1. HNE Adduct Accumulation and γH2AX Expression in Skin Fibroblasts from UV-A- Exposed Hairless Mice

**Figure 1:**
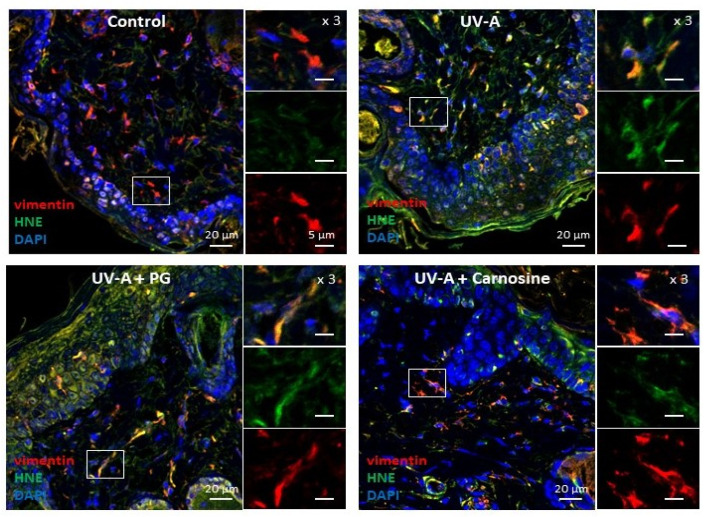
Expression of HNE adducts in fibroblasts in the skins of UV-A-exposed hairless mice. Immunofluorescence pictures (and higher magnification ×3 of each staining on the right of each picture) showing the presence of vimentin in fibroblasts (red), HNE adducts (green), and the merge (yellow). Upper left panel, control (untreated); Upper right panel, UV-A irradiated (20 J/cm^2^/d up to 600 J/cm^2^); lower left panel, propylene glycol (PG)-treated UV-A irradiated; lower right panel, carnosine (1% in PG)-treated/UV-A irradiated, as indicated in [32]. Scale bar, 20 µm, magnification 5 µm. Nuclei were stained with DAPI (blue). These data are representative of five separate experiments.

Skin samples from UV-A irradiated hairless mice [32] were used to investigate the presence of senescence patterns in dermal fibroblasts, together with the formation of HNE adducts in these cells. As previously reported [32], immunofluorescence and confocal imaging experiments pointed out the presence of HNE adducts throughout the skin, including in dermal fibroblasts identified by vimentin staining (Figure 1).

As shown in Figure 1, HNE adducts could colocalize with vimentin in fibroblasts, consistently with previous reports showing a sensitivity of this intermediate filament protein to lipoxidation, carbonyl, and electrophilic stress [35], as well as glycation and AGEs in senescent human fibroblasts [18], HNE, and MDA [35,36,37].

**Figure 2:**
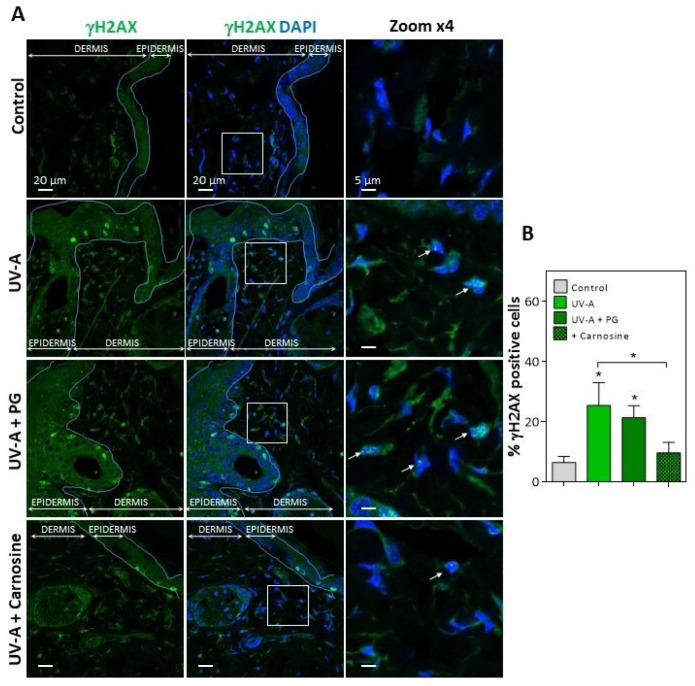
Expression of γH2AX in the skins of UV-A-exposed hairless mice. (**A**), immunofluorescence and confocal imaging of γH2AX (green) in skin fibroblasts from hairless mice untreated (control), UV-A-exposed (UV-A), UV-A-exposed/propylene glycol (UV-A+PG), and UV-A-exposed + carnosine (1% in PG). Nuclei were stained with DAPI (blue). Scale bar, 20 µm. Inserts indicate the area selected for higher magnification ×4, right panels. White arrows indicate the γH2AX positive nuclei. (**B**), statistical quantification of the percentage of γH2AX positive cells in the dermis area. The data are expressed as medians ± interquartile range. Statistical significance was assessed using the nonparametric Mann-Whitney U test (* *p* < 0.05).

In UV-A-exposed skins, the presence of HNE adducts was associated with an increased expression of γH2AX foci (Figure 2), which characterize the formation of DNA double-strand breaks and DNA repair site(s), in response to cytotoxic agents or in senescent cells [16,38]. Interestingly, skins from mice preventively treated by carnosine [32] showed much lower levels of vimentin modification by HNE, and a reduced number of γH2AX positive cells (Figure 1 and Figure 2), suggesting that the protective effect of carnosine on skin photoaging involved a prevention of fibroblast senescence. It is of note that the carnosine vehicle, polyethylene glycol (PG), did not protect against the formation of HNE adducts on vimentin and the expression of γH2AX positive cells (Figure 1 and Figure 2). Since carnosine is a potent carbonyl and HNE-scavenger [33,34], it can be hypothesized that HNE generated by UV-A radiation contributes to fibroblast senescence in photoaged skins.

Then we checked whether HNE either freely added to the cultured medium of murine fibroblasts, or generated in these cells by UV-A, may generate the expression of senescence markers and the formation of adducts on vimentin, and whether these responses could be prevented by carnosine.

### 3.2. HNE- and UV-A-Exposed Skin Fibroblasts Exhibit Senescence Patterns

**Figure 3:**
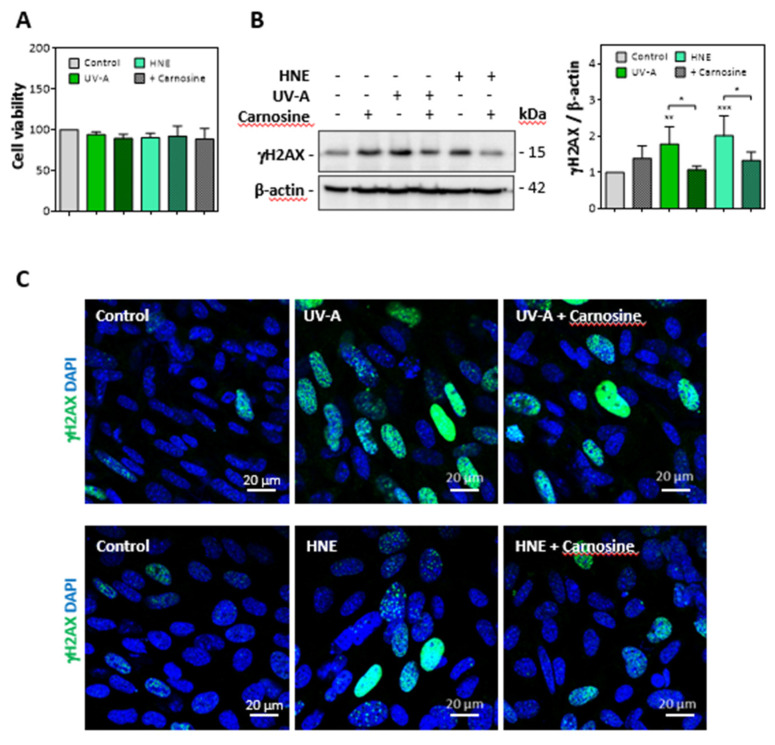
Expression of γ-H2AX evoked by UV-A or HNE in fibroblasts nuclei. Fibroblasts were exposed to UV-A (two cycles of irradiation, each up to 15 J/cm^2^ in HBSS medium) or HNE (30 µM, 4 h in HBSS medium), ± carnosine (100 µM). (**A**), Cell viability evaluated by the MTT test, in fibroblasts exposed to UV-A or HNE, ± carnosine. (**B**), expression of γH2AX and protective effect of carnosine evaluated by western blot of fibroblasts exposed to UV-A or HNE, as described in 3A. Right panel, statistical quantification of γH2AX, expression, data are represented by means ± SEM of four independent experiments. * *p* < 0.05; ** *p* < 0.01; *** *p* < 0.001. (**C**), immunofluorescence and confocal imaging of γH2AX foci in fibroblasts treated by UV-A (upper panels) or HNE (lower panels) ± carnosine. Nuclei were stained with DAPI. Scale bar, 20 µm.

Cultured murine skin fibroblasts were exposed to mild UV-A radiation (two exposures in 48 h, each up to 15 J/cm^2^), and the expression of senescence parameters was analyzed together with the modification of vimentin by HNE adducts. Alternatively, we checked whether skin fibroblasts incubated with HNE (30 µM, 4h in HBSS) exhibit cellular senescence and vimentin modification. No significant toxicity was observed under the used conditions (Figure 3A).

As shown in western blots of cell extracts, the expression of γH2AX was strongly increased in fibroblasts exposed to UV-A or incubated with HNE (Figure 3B). Likewise, confocal imaging pictures (Figure 3C) confirmed the increased expression of γH2AX in fibroblasts either challenged with HNE or exposed to UV-A. In these experiments, the protective effect of carnosine on both UV-A and HNE treatments supported a role for HNE in UV-A-induced γH2AX expression in fibroblasts (Figure 3B,C).

We then checked whether UV-A and HNE may affect the activity of sirtuins and the turnover of acetylated proteins. Sirtuins, and especially SIRT1, which is the most characterized sirtuin in mammalian cells, are nicotinamide dinucleotide (NAD+)-dependent deacylases, which play an essential role in the prevention of senescence, by stabilizing the chromatin structure and by deacetylating histones, transcription factors, and DNA repair proteins [39,40]. As sirtuins are thought to play an important role in skin photoaging, UV-A and UV-B-induced damages and oxidative stress responses [41], we checked whether HNE may alter SIRT1 expression and activity in UV-A-exposed fibroblasts.

**Figure 4:**
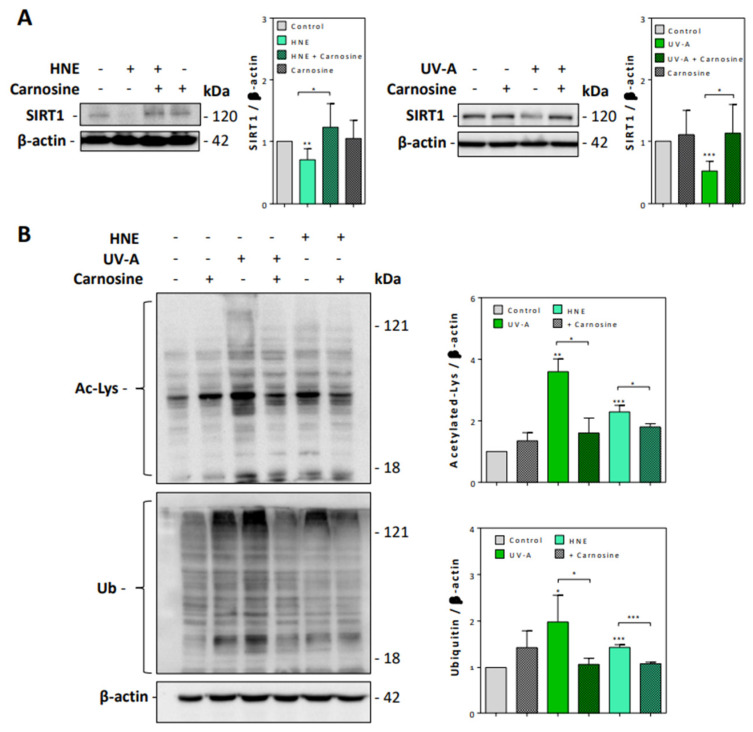
SIRT1 expression and accumulation of acetylated and ubiquitinated proteins in UV-A- and HNE-treated fibroblasts. (**A**), SIRT1 expression evaluated by western blot in fibroblasts exposed to UV-A or HNE, and protection by carnosine, using β-actin as control. Left panel, effect of HNE; right panel, effect of UV-A. On the right of each western blot picture, statistical quantification of SIRT1 in UV-A- or HNE-treated cells vs control untreated fibroblasts. (**B**), Western blot experiments showing the accumulation of acetylated (upper panel), and high molecular weight polyubiquitinated proteins (lower panel), in fibroblasts stimulated by HNE or UV-A, and protective effect of carnosine. Right panel, statistical quantification of acetylated and ubiquitinated vs control untreated fibroblasts. These results are a mean of four separate experiments and are expressed as means ± SEM. Statistical analysis was assessed using a Student *t*-test. * *p* < 0.05; ** *p* < 0.01; *** *p* < 0.001.

As shown in Figure 4A, SIRT1 levels were strongly decreased in fibroblasts exposed to UV-A or HNE, in correlation with the accumulation of acetylated proteins (Figure 4B). Carnosine pretreatment restored the expression of SIRT1 and prevented the accumulation of acetylated proteins in both UV-A- and HNE-treated fibroblasts.

Likewise, the exposure to UV-A or HNE induced an accumulation of ubiquitinated proteins (Figure 4B), in agreement with previous studies showing an accumulation of polyubiquitinated and oxidized proteins upon UV exposure and in human senescent fibroblasts [42,43,44]. Carnosine prevented the accumulation of ubiquitinated proteins in both UV-A-exposed and HNE-stimulated fibroblasts (Figure 4B).

### 3.3. HNE Adduct Formation on Vimentin in Fibroblasts Exposed to UV-A Radiation

**Figure 5:**
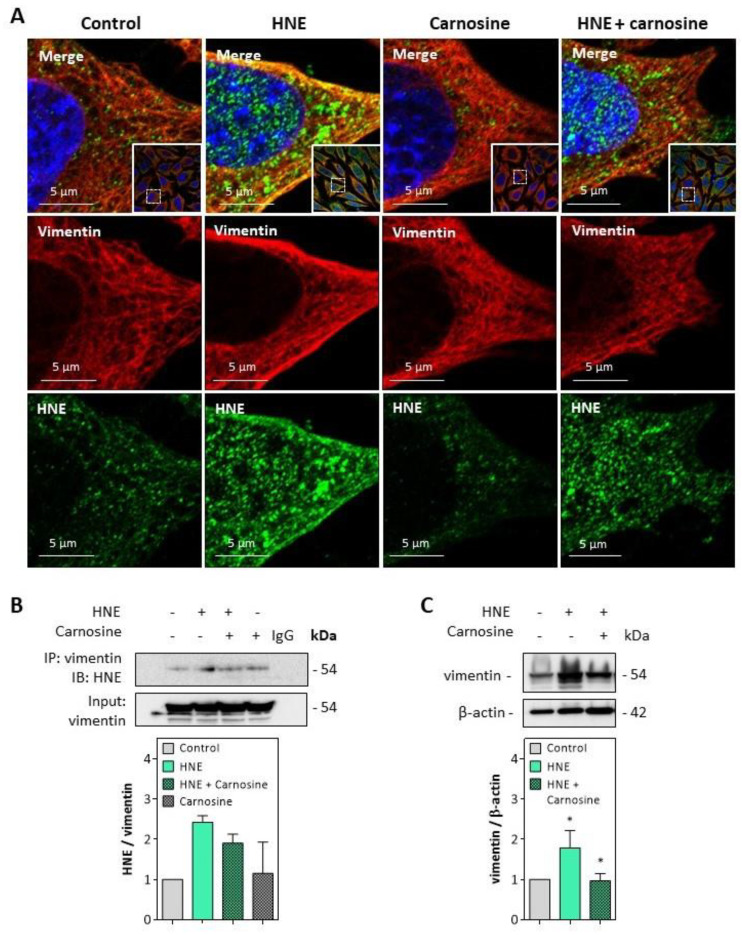
HNE adduct formation on vimentin in fibroblasts exposed to HNE. Cultured fibroblasts were exposed to UV-A (two cycles of irradiation, each up to 15 J/cm^2^ in HBSS medium) or HNE (30 µM, 4 h in HBSS medium), ± carnosine (100 µM). Western blot analyses were carried out on cells incubated with HNE as described above, followed by 24 h incubation at 37 °C in fresh RPMI medium supplemented with 1% FBS as indicated in the Method section. (**A**), Representative confocal imaging pictures showing the presence of HNE adducts (green, lower panel) on vimentin (red, middle panel), and the merge (yellow, upper panel), in fibroblasts incubated with HNE, and protective effect of carnosine. Nuclei were stained with DAPI (blue). Scale bar, 5 µm. In insert, original 63× confocal image. (**B**), Detection of HNE adducts on vimentin immunoprecipitates from fibroblasts incubated with HNE and protective effect of carnosine (mean of two separate experiments) (**C**), Expression of vimentin in fibroblasts incubated with HNE and protective effect of carnosine. These results are the mean of three separate experiments and are expressed as means ± SEM. Statistical analysis was assessed using a Student *t*-test. * *p* < 0.05.

Immunofluorescence and confocal imaging experiments carried out on fibroblasts incubated with HNE (30 µM, 4 h) pointed out the formation of HNE adducts on vimentin, particularly at the cell membrane (Figure 5A). These data fit with previous observations reported by Frescas et al. for MDA [37], suggesting that membrane-bound MDA-vimentin could be a mechanism allowing the eradication of senescent cells by humoral innate immunity.

The formation of HNE adducts on vimentin was confirmed on vimentin immunoprecipitates of fibroblasts incubated with HNE (Figure 5B). Moreover, an increased expression and slight fragmentation of vimentin filaments were observed upon incubation with HNE (Figure 5C), in agreement with previous reports from Perez-Sala group, with HNE diamide [35,36]. Vimentin modification and fragmentation were prevented by carnosine (Figure 5A–C).

**Figure 6:**
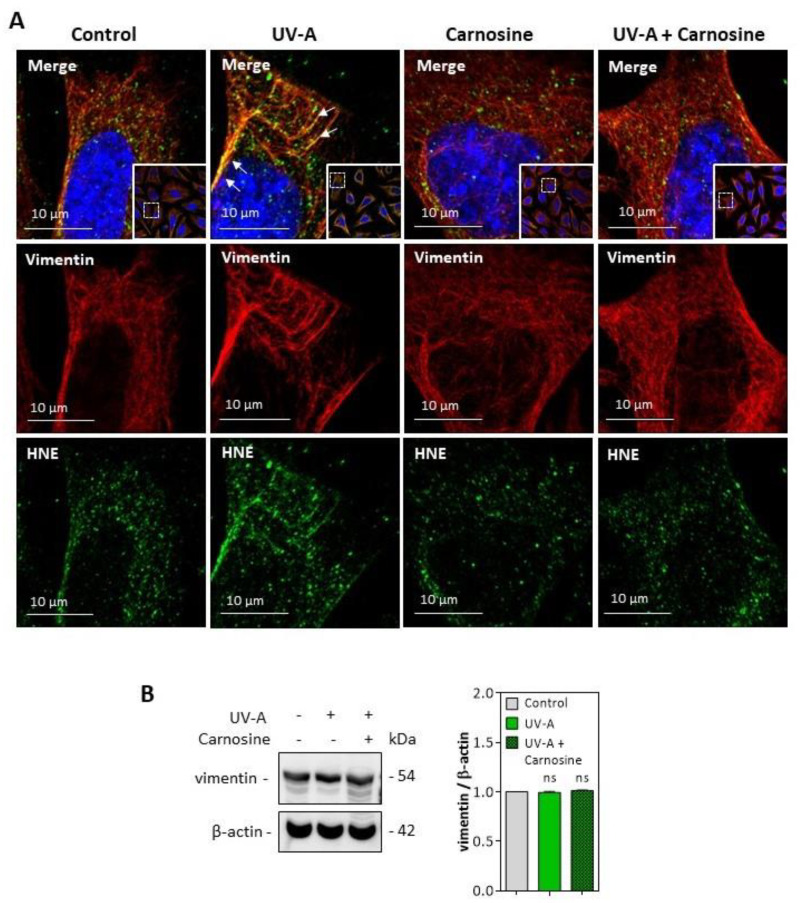
HNE adduct formation on vimentin in UV-A-exposed fibroblasts. (**A**) representative confocal imaging pictures showing the presence of HNE adducts (green, lower panels) on vimentin filaments (red, middle panels), and the merge (yellow, upper panel), in fibroblasts exposed to UV-A ± carnosine. White arrows indicate the colocalization areas (merge pictures). Nuclei were stained with DAPI (blue). Scale bar, 10 µm. (**B**)**,** Expression of vimentin in fibroblasts exposed to UV-A, and effect of carnosine. These results are the mean of three separate experiments and are expressed as means ± SEM. Statistical analysis was assessed using a Student *t*-test. ns, non-significant.

HNE adducts were detected on the vimentin filament network of UV-A treated cells (Figure 6A), though no fragmentation of vimentin was observed in the used experimental conditions (Figure 6B). Again, the preincubation of fibroblasts with carnosine completely prevented the modification of vimentin evoked by UV-A.

Altogether, these results indicate that HNE and UV-A radiation trigger a modification of vimentin filaments in cultured skin fibroblasts, which could be prevented by carnosine, consistent with its protective effect on the expression of senescence markers in these cells.

## 4. Discussion

In this article, we show that HNE triggers the expression of senescence patterns in cultured fibroblasts, either directly or when generated by UV-A radiation. The presence of HNE adducts on vimentin in dermal fibroblasts from skins of hairless mice exposed to UV-A, together with γH2AX foci as markers of senescence, suggests that HNE plays a role in fibroblast aging. This role was also supported by the protective effect of carnosine on vimentin modification and the expression of senescence markers in cultured fibroblasts and in the skins of hairless mice.

Lipid peroxidation products are rapidly generated upon skin exposure to oxidative stress generated by UV-A radiation [9,32,45,46,47,48,49]. Lipid peroxidation-derived aldehydes rapidly react with nucleophilic groups such as free amino group of lysine, sulfhydryl group of cysteine, and imidazole group in histidine [21,22,23], to form adducts that accumulate on proteins and modify their function. We previously described the presence of HNE adducts on altered elastin fibers in the skin of hairless mice exposed to UV-A, suggesting a role for lipid peroxidation in the development of actinic elastosis lesions [32]. We show here that HNE adducts generated by UV-A in fibroblasts are detected on vimentin, a cytoskeletal protein sensitive to oxidants and electrophiles, and a target of glycation and AGEs, with possible implication in fibroblast senescence [19,20,35,36].

Vimentin plays an important role in cell motility, orientated cell migration, and wound healing, by controlling actomyosin contraction forces and cellular interactions with ECM [20,50]. Monico et al. recently reported the consequences of vimentin modification on fibroblast motility [36]. The post-translational modification of vimentin by electrophilic aldehydes may severely alter the intermediate filament network, causing a loss of contractile capacity of fibroblasts depending on the extent of oxidative stress, as observed in the aging process. Likewise, an increased expression of vimentin could be observed in senescent fibroblasts [51]. Our results show a strong expression of HNE-modified vimentin in the cell membrane, in agreement with the senescence phenotype reported by Frescas et al. [37]. As suggested by this group, the modification by MDA of the senescence-associated cell-surface vimentin could serve as an “eat me” signal, allowing the phagocytosis of senescent cells by macrophages. This mechanism could become impaired with age, resulting in an accumulation of senescent cells [37]. At the cellular level, the modification of vimentin by oxidants and electrophiles results in the disruption of the intermediate filament network and the generation of intracellular aggresomes [52]. In our study, we observed a slight fragmentation of vimentin in fibroblasts incubated with HNE, in agreement with Monico et al., who recently showed that vimentin oxidation or electrophilic modification, results in the disruption of the vimentin filament network, with possible pathological consequences in aging [36]. Likewise, several AGEs, including carboxymethyl lysine, carboxyethyl lysine, or pentosidine, may modify vimentin, and generate its dysfunction and accumulation in aggresomes, including in the skin [18].

The modification of vimentin by HNE was correlated in vitro and in vivo with the expression of senescence markers, such as an increased number of cells positive for γH2AX [16]. γH2AX is a molecular aging marker corresponding to the phosphorylated form of H2AX histone on S139, occurring in response to DNA damages and DNA double-strand breaks (DSB) [16]. UV-B and-C may promote the formation of γH2AX independently of DSB, possibly via an intervention of nucleotide excision repair factors that could expose the phosphorylation site on H2AX [53]. In contrast, the mechanisms by which UV-A triggers DNA damage are less clear and may involve cellular photosensitizers and oxidative stress, leading to DSB formation and H2AX phosphorylation [53]. A role for HNE could be expected in the accumulation of γH2AX foci in UV-A-exposed hairless mice and in cultured fibroblasts, in view of its genotoxic properties and its ability to trigger H2AX phosphorylation as reported [53,54] and as observed in our study in fibroblasts incubated with HNE. This role of HNE was also emphasized by the protective effect of carnosine, a histidine dipeptide, exhibiting mild antioxidant properties, and a high efficacy for neutralizing HNE via its imidazole moiety, allowing to prevent protein modification and dysfunction evoked by HNE adducts [33,34,55]. Carnosine prevented the modification of vimentin by HNE and the accumulation of γH2AX foci in fibroblasts from UV-A-exposed mice. Furthermore, carnosine also reduced the senescence signaling evoked by UV-A, pointing out a role for HNE in the mechanism of photoaging.

UV-A and HNE promoted the accumulation of ubiquitinated proteins which are characteristic of the fibroblast aging process, either physiological or promoted by UV exposure [42,43,44]. Carnosine limited the accumulation of ubiquitinated proteins in UV-A-exposed fibroblasts, as reported for antioxidants such as quercetin, which may increase the lifespan and viability of human fibroblasts by stimulating proteasome activity [56]. Likewise, carnosine restored the expression of SIRT1 and reduced the accumulation of acetylated proteins, which were altered by UV-A and HNE treatment. Sirtuins play a key role in the regulation of cell homeostasis and could prevent skin photoaging [39,40,41]. Their activity is highly regulated by oxidative stress, either increased by mild redox variations or inhibited by high and prolonged oxidant conditions, particularly post-translational oxidative modifications resulting in sirtuin dysfunction and inhibition of their deacetylase activity [57]. This is of particular importance for SIRT1, which is highly activated by mild oxidative stress to promote antioxidant responses and mitochondrial biogenesis, via the diacylation of redox-sensitive transcription factors [57,58]. In contrast, high oxidative stress conditions and the modification of thiols residues by aldehydes decrease the activity and expression of SIRT1, leading to an accumulation of acetylated proteins [58,59]. Importantly, SIRT1 expression and activity are decreased in senescent cells, and SIRT1 pharmacological inhibition promotes a premature cellular senescence [59]. In our study, the mechanism by which HNE and UV-A elicited a decrease in SIRT1 expression was not investigated, but could involve the formation of HNE adducts on SIRT1, and its subsequent proteolytic degradation. This was reported in cardiomyocytes from aged mice, in which a decreased expression of SIRT1, associated to its modification by carbonyl stress, could contribute to myocardial ischemic intolerance [60]. Carnosine restored SIRT1 expression and reduced the accumulation of acetylated proteins in fibroblasts, probably via the neutralization of HNE generated by UV-A, pointing out the potent anti-aging properties of this agent.

## 5. Conclusions

In conclusion, these results provide new insight in the implication of HNE in photoaging and emphasize the potent efficacy of carnosine in preventing vimentin modification, fibroblast senescence and more generally photoaging of UV-A-exposed skins, via its carbonyl-scavenger properties.

## Data Availability

Data are contained within the article.
